# Promoting the Implementation of Co‐Produced Cochrane Evidence: An Exploratory Study of Improving Partnering With Consumers

**DOI:** 10.1002/cesm.70071

**Published:** 2026-02-03

**Authors:** Bronwen Merner, Louisa Walsh, Janet Jull, Nora Refahi, Vasileios Tsialtas, Benjamin Shemesh, Mel Kotze, Rebecca Ryan

**Affiliations:** ^1^ Department of Public Health, Cochrane Consumers and Communication, Centre for Health Communication and Participation La Trobe University Melbourne Victoria Australia; ^2^ Department of Nursing and Allied Health Swinburne University of Technology Hawthorn Australia; ^3^ Department of Health Sciences Carleton University Ottawa Canada; ^4^ Western Health Melbourne Australia; ^5^ Better Health Network Melbourne Australia; ^6^ Safer Care Victoria Melbourne Australia; ^7^ Austin Health Melbourne Australia

**Keywords:** Cochrane, co‐production, health services decision‐making, implementation, patients

## Abstract

**Introduction:**

Co‐production of evidence syntheses has the potential to facilitate translation of research findings into policy and practice. However, few studies have explored the process of implementing co‐produced evidence. This gap limits our understanding of how, and to what extent, co‐production promotes knowledge translation.

In this study, we used an implementation science lens to explore factors influencing the implementation of the Best Practice Principles in partnering with consumers (BPP) in hospitals in Melbourne, Australia. The BPP were developed as part of a co‐produced Cochrane qualitative evidence synthesis exploring consumers' and health providers' experiences and perceptions of partnering. We use the findings of our study to develop strategies for evidence synthesis teams engaged in co‐production to optimize the implementation of their review findings.

**Methods:**

This exploratory, qualitative study was informed by cooperative inquiry and normalization process theory (NPT). A six‐member panel, including researchers, policy makers and consumers, guided data collection and analysis. Data collection involved semi‐structured interviews with eleven participants (including consumer engagement leads, consumer representatives, and a policymaker) about how to implement the BPP in Melbourne hospitals. Interviews were analyzed using framework analysis.

**Results:**

Interview participants reported the BPP were relevant to practice, consumer‐centered, practical, and flexible. There were several additional factors that could impact their uptake into practice. These included integration of the BPP into government policies and guidelines, evidence of the cost/benefit of BPP implementation, endorsement from health service leadership, involvement of consumers throughout the implementation process, a structured implementation, and flexible measurement of implementation success.

**Conclusion:**

This exploratory study suggested that the BPP, a tool developed through a co‐produced Cochrane qualitative evidence synthesis, promoted knowledge translation. Other factors at the macro‐ (political and economic), meso‐ (systems and organizations), and micro‐ (individual) levels could influence the implementation's success. Implications for evidence synthesis teams aiming to optimize the knowledge translation of their review results are discussed.

## 1 Introduction

Co‐production of evidence syntheses has the potential to facilitate knowledge translation into practice [1]. According to the United Kingdom's National Institute for Health and Care Research (NIHR), co‐production is “an approach in which researchers, practitioners and members of the public work together, sharing power and responsibility from the start to the end of the project, including the generation of knowledge” [2]. Compared to traditional methods, co‐produced research is proposed to lead to significant social impacts including knowledge that is more relevant to practice [3, 4]. As Concannon (p. 990) states, “perhaps the most important benefit of an active stakeholder engagement program is its potential to move research evidence off of bookshelves and into practice” [5]. Few studies have described the process of implementing such evidence. This gap limits our understanding of how, and to what extent, co‐produced evidence promotes successful knowledge translation. As a result, the extent to which co‐produced evidence can facilitate knowledge translation is uncertain for evidence synthesis teams.

In 2023, we (B.M., R.R., L.W., N.R.) published a Cochrane qualitative evidence synthesis (QES) titled *Consumers' and health providers' views and perceptions of partnering to improve health services design, delivery, and evaluation* [6]. The QES was co‐produced with an 18‐member interest‐holder panel of consumers, health practitioners and policymakers [7]. During the review, we developed a set of best practice principles (BPP) for partnering with consumers in health services based on the review findings. The panel requested that the BPP be developed from the review findings, to ensure these were easier to adopt in practice. A summary of the BPP is shown in Table 1.

**Table 1:** Best practice principles for partnering with consumers (shortened version).

Principle	Description	Selected examples of best practice
Leadership and health service culture	Partnering is endorsed by government interest holders and health service leaders.	Partnering is endorsed through government policies and standards relevant to service design, delivery and evaluation Government supports the implementation of partnering policies and standards in health organizations through guidance and funding Health service leaders embed partnering across their whole organization
Diversity	Partnering opportunities are open to a diversity of consumers.	Resources are dedicated to recruiting consumers from underrepresented groups Meeting times and arrangements are flexible Consumers are supported to participate through means including remuneration, reimbursement of expenses and the provision of other resources (e.g., childcare, accessible venues, access to interpreters, support for people with communication disabilities)
Equity	Consumers have the support they need to contribute equitably to partnering. Power imbalances between health providers and consumers are addressed.	Consumers are involved in recruiting new consumers for partnerships The proportion of consumers to health providers in partnerships is equitable Some consumers may prefer smaller, less formal meetings Consumers have access to consumer‐only meetings, peer networks and data (such as consumer feedback surveys) to support their participation in partnering Consumers are offered ongoing training relating to their role, working in partnership and the health service
Mutual respect, shared vision, and regular communication	Consumers and health providers respect each other for their expertise and knowledge. The purpose of the consumer role is explicit to both consumers and health providers. They share a common vision for partnering, and foster commitment and trust. Consumers and health providers exchange information, communicate regularly and maintain strong links with health service leadership.	The chairperson is impartial and fosters the development of a shared vision for the partnership Consumers participate in shaping their role and/or are clear about role expectations Health providers receive training about how to partner with consumers, including the scope of the consumer role and the value of consumers' contributions Employment of a consumer coordinator may help to facilitate support of consumers' views with health service leadership
Shared agendas and decision‐making	Consumers and health providers set agendas jointly and share decision‐making.	Consumers may lead or co‐lead a partnership Token involvement in agenda‐setting is avoided, such as health providers determining the agenda without consumer input, assuming consumers do not want to be involved in particular agenda items or refusing to countenance consumer contributions perceived to be outside the health service's priorities Token involvement in decision‐making is avoided, such as consumers being asked to “rubber‐stamp” decisions already made, and consumers not being included in key discussions
Influence	Consumers contribute to tangible improvements in health service policy and delivery.	Consumers' views and suggestions are listened to and followed up
Sustainability	Consumers and health providers involved in partnering may experience positive or negative impacts of their involvement over time. Negative impacts should be addressed to facilitate the sustainability of the partnership.	Examples of impacts for consumers:
		Positive impacts: increased knowledge, skills and confidence Negative impacts: difficulty sustaining involvement over long periods, managing the impact of illness while participating in partnerships
		Examples of impacts for health providers:
		Positive impacts: benefit from consumers' unique perspectives Negative impacts: insufficient time to participate in partnering, perceiving they will not be able to meet consumers' expectations

*Note:* Adapted from Merner et al. consumers' and health providers' views and perceptions of partnering to improve health services design, delivery, and evaluation: a co‐produced qualitative evidence synthesis. *Cochrane Database of Systematic Reviews 2023*, Issue 3. Art. No.: CD013274. DOI: 10.1002/14651858.CD013274.pub2.

After publishing the QES, we sought to explore how the BPP could be implemented into hospitals in Melbourne, Australia. Given that the BPP had been co‐produced by an Australian interest‐holder panel and was based on gold standard evidence, we anticipated that the principles had the potential to effect tangible improvements in partnering with consumers in Australian health services. Therefore, we conducted an exploratory study to understand the context for implementing the BPP, informed by implementation science. We used these findings to develop strategies for evidence synthesis teams engaging in co‐production to optimize the implementation of their review findings.

## 2 Materials and Methods

### 2.1 Methodological Orientation and Theory

This exploratory, qualitative study used a cooperative inquiry research design informed by normalization process theory (NPT) [4, 8, 9].

#### 2.1.1 Cooperative Inquiry

Cooperative inquiry is an approach to co‐production in which people collaborate to define research questions and methodology [4]. In this study, a Melbourne‐based panel of four researchers, two consumers, two hospital representatives, and two policy makers engaged in cooperative inquiry to (1) guide data gathering to address the project aim, and (2) to analyze and derive meaning from the data gathered. Three of the researchers and one consumer member were co‐authors on the Cochrane QES that developed the BPP. The panel was purposefully recruited through the research team's (B.M., L.W., R.R.) professional networks and met three times via Zoom over the project (May 2023 to February 2024). Consumer representatives were remunerated $120 AUD/meeting for their involvement.

Researcher panel members (B.M., L.W., J.J., R.R.) drafted a research proposal including the project aims, methods, and outcomes. In the first panel meeting, the project aims were discussed and clarified. For example, the panel advised that an implementation plan be produced once data collection and analysis had been undertaken. The implementation plan, co‐developed with the panel and drawing from the Cochrane Effective Practice and Organization of Care Group's intervention taxonomy, is shown in S1 [10].

#### 2.1.2 NPT

NPT was selected as the implementation science framework for this study because it explores how implementing a new practice, such as the BPP, can improve partnerships and become a routine part of everyday work [8, 11]. NPT also allows exploration of the process of implementation and how change could occur, rather than focusing solely on whether the implementation would achieve specific outcomes [12]. NPT posits that the act of embedding a new practice is promoted or inhibited through the following four mechanisms:

1. Coherence‐building: how people make sense of the practice.
2. Cognitive participation: how people are enrolled in implementation.
3. Collective action: the work of enacting the practice.
4. Reflexive monitoring: how people assess its success [9].

With cooperative inquiry, NPT informed the data collection and analysis methods.

### 2.2 Data Collection

During the panel's second meeting, it was agreed that the panel would decide the data collection method used for the project [2]. To identify potential methods, the researcher panel members identified two potential methods for gathering data to address the research question (qualitative survey or interviews). Other panel members were emailed a written summary of the advantages and disadvantages of each method and asked to vote for their preferred option; semi‐structured interviews received the most votes. Informed interest‐holder voting has been used previously in this way to determine the research method [2]. The majority of the panel voted for semi‐structured interviews because of the method's potential to gather in‐depth data.

Interview participants were purposively sampled from the following groups of interest‐holders that would be involved in or affected by implementation of the BPP: consumer representatives (patients, carers, or family members), consumer engagement leads (hospital representatives who promote and support consumer engagement in their health service), and government policy makers.

Participants were recruited through the panel's professional networks. They were emailed an invitation to participate which included a description of the study and a copy of the BPP. Willing participants completed a consent form in REDCap prior to their interview [13]. Cooperative inquiry methodology allows people to take on both participant and researcher stances [4]. Therefore, panel members could also be interviewed on request. Panel members who agreed to be interviewed were also required to formally consent to participate.

The interview guide (see S2) was developed with feedback from the panel and informed by NPT. The interview guide was not formally piloted, but changes were made over the course of the interviews to ensure sufficient data was collected about all aspects of NPT. Interviews were conducted by B.M., a female researcher who has a PhD in health services research and experience conducting qualitative interview studies on a range of health‐related topics. Interviews were audio‐recorded and transcribed verbatim. During the interview, B.M. kept written notes about topics raised by the participant that could be probed in follow‐up questions. Transcripts were not member‐checked due to time constraints.

### 2.3 Data Analysis

Transcripts were analyzed by B.M. using framework analysis in NVIVO 2015 [14, 15]. The coding framework consisted of the four NPT mechanisms. First, data was coded to the appropriate mechanism/s. Second, data within each mechanism was compared and contrasted and categorized into themes and sub‐themes. Themes were refined until the resulting framework incorporated all interview data. These draft themes and sub‐themes were circulated to the researcher team (R.R., J.J., L.W.) for feedback. Any inconsistencies in the analysis or lack of supporting evidence for claims were identified and addressed before the results were sent to the panel. During the final panel meeting, members provided feedback about potential gaps in the analysis. Any identified gaps were reviewed against the data set by B.M., and refinements were made to the analysis when appropriate.

## 3 Results

### 3.1 Characteristics of the Sample

Ten interviews were conducted via Zoom with 11 participants (three of them panel members) in November and December 2023 (two panel participants were interviewed within the same session). Interviews lasted approximately 45 min each. No participants withdrew during the study.

As shown in Table 2, participants comprised six consumer engagement leads, four consumer representatives, and one policy maker. A range of hospitals (both specialist and generalist) across Melbourne were represented in the sample. No health practitioners were recruited within the time available for data collection, and therefore, it is unlikely that data saturation was achieved. However, given that other participants were sampled from a range of hospital settings, it is likely that some of their experience of consumer partnerships and organizational knowledge would be similar to those of health practitioners working in the same hospital.

**Table 2:** Interview participants' characteristics.

Characteristic	Number of participants
Gender	
Female	9
Male	2
Type of hospital	
Specialist hospital	4
Generalist hospital	6
Not from a hospital (e.g., policymaker)	1
Location of hospital	
West	2
Inner West	4
Inner East	1
Inner South	1
East	1
South	1
Not from a hospital (e.g., policymaker)	1
Role	
Consumer representatives	4
Consumer engagement leads	6
Policymakers	1

### 3.2 Perceived Facilitators for Improving the Implementation of the BPP

Table 3 summarizes the perceived facilitators, with illustrative quotes, that would improve implementation of the BPP.

**Table 3:** Perceived facilitators to improve the implementation of the BPP and illustrative quotations.

Implementation mechanism	Facilitator	Illustrative quotations
Coherence building	Promote the principles as practical, consumer‐centered, and relevant	… like this area [of partnering with consumers] … it's very fluff[y… Whereas [with the BPP examples], you need to provide evidence that you've done this, this [and] this, because this is best practice. P1, consumer engagement lead
		I looked at [the BPP] and I was like, “Yes, this is brilliant. This is fantastic.” Because it cements everything that we know to be true about partnering and it just feels right. Like these are things that we say, “You really want this. You either want to be working on it, or you want it right when you are engaging with consumers.” So it was certainly a tool that I was like, “Yes, this is really great.” P5, consumer engagement lead
		There is a great need for [the BPP] because a lot of [consumers] would benefit from having something like this behind them. Because then we're all sort of working in a framework that says basically, “This is a safe space and you can ‐ we've all got an equal vote.” P10, consumer representative
	Champion the BPP as useful for all health services, regardless of current partnering practices	Depending on the maturity of community engagement or partnership within an organisation, [the BPP] can either benefit a lot or benefit not so much and just be a reminder or a little tap on the shoulder. P5, consumer engagement lead
		For some people, [the BPP] are an affirmation of some things that they already do and that gives them better confirmation and affirmation and I guess some support to continue doing those. For some, obviously, who are the start of their journey, or just grappling with the concept of what partnering means, then they might be a bit daunted by the breadth and the “spreadth” and also the depth of what really is required. So it depends really, I guess, on the gradient of partnering where people are starting at. But I think that for me, I feel we have the capacity and it's our responsibility to really work hard at implementing those and raising the bar of expectations. P11, policymaker
Cognitive participation	Integrate the BPP into government policies or quality standards	Yes, my guess would be that [having government drive the implementation] would impact in how [the BPP are] implemented because if… the hospital… has to provide work, it has to work, you know? It will give that work value. It will value that work. P9, consumer representative
		It would probably have to come from like the Commission [Australian Commission on Safety and Quality in Health Care], Safer Care Victoria; it's gotta be a high‐level governing body, I think. Make it a part of Standard 2 [an accreditation standard] ‐ there you go! P1, Consumer engagement lead
	Provide evidence that the benefits of the BPP outweigh implementation costs	… That's still a tension in terms of implementation: the time factor and the resources. It's not that people will disagree with it, it's about the time and the effort and the length of time to show results, and how you attribute those results to the fact that there's better partnering in decision‐making or people have more access to information or decisions… P11, policymaker
	Involve health service leaders	If [the implementation] is driven by the Board, like you're going to get far more… it's going to be a lot easier. P1, consumer engagement lead
		So operationally, person in my role to make sure that it is out there and people are engaging with it. But definitely needs support from my director, but also her director on top of that, which we sit under clinical operations. P8, consumer engagement lead
		Yes, that if there is no ‐ how do you say? ‐ genuine understanding by the executive of any institution, and the executive is run by the personality of the CEO. So if the executive is a bit afraid of the CEO, or getting in the wrong side of the CEO, or getting the wrong side of the Department of Health, there is the element that it's going to affect the relationship in how they view the consumer, or how they view the advocate. So this is absolutely sine qua non, I know. P9, consumer representative
	Designate (and fund) key implementation facilitators	There's a lot of what's in [the BPP] that would be driven by my team, by consumer engagement. So, a lot of that sort of stuff would sit with us. P2, consumer engagement lead
		Interviewer: How easy do you think it would be to integrate some of the content of the BPP into those different resources?
		P1 (Consumer engagement lead): [Laughs] I laugh because in theory, exceptionally easy. But again, it comes back to that resourcing challenge and what is the most pressing thing to get done, essentially? That would be really the key barrier.
	Involve and support consumers throughout the implementation but do not burden them	And most definitely we should be part of the whole process. And I ‐ well we're the ones that it's about anyway, so I think it's really important ‐ whether from the beginning, right through to the end, in any setting where there's policies and procedures happening, we should be there as well. P6, consumer representative
		I think it would be hard for a consumer to lead [the BPP implementation] unless they were employed as a lived experience consumer within the organisation, and that would be part of their portfolio. But that doesn't happen yet. One day hopefully. P5, consumer engagement lead
		I think that's [the hospital's] responsibility. I think our role is basically to voice our concerns and the things that are important to us. P7, consumer representative
	Promote the BPP across the health service	But unless people are talking about it ‐ word of mouth, it's really, really important. You need the people that are on the ground that are working with others to say, “Have you heard about this? This is really important.” Obviously hit your ‐ hit some people which are already committed to the cause and using examples to be able to, “This is best case scenario,” or, “This is what it means or what it looks like when it's done well.” Those people don't see ‐ don't make those excuses that we're so used to hearing. P8, consumer engagement lead)
Collective action	Structure the implementation and ensure it adds value for partnership members	It can't be willy nilly, it's got to be a structured approach‐ this is how we do it. And then you can provide that evidence should accreditation come, your snap accreditation‐ yes, this is exactly how we do it, we follow the [BPP]. P1, consumer engagement lead
	Support partnership members to implement the BPP	I guess user friendly, easy to read, easy to adopt, consultation, already developed pamphlets, information, strategies in how they are used… I think you'd want a whole package. You'd want a whole suite. P5, consumer engagement lead
		There is ‐ there also one of the things that one of my biggest ‐ I think one of the biggest barriers for consumers, consumers are not properly supported. If consumers have any conflict for whatever reason ‐ for the color of the sky today ‐ with the management, there is no way of redressing, the consumer is basically leaves ‐ as I have seen, leaves. P9, consumer representative
	Integrate the BPP into internal policies and procedures	I think if the [BPP] were aligned to the organisational… then it would be hard not to go, “Well these are our guiding principles and policies. You should be doing all of this.” P5, consumer engagement lead
		And I like what you said there, [interviewer], sustainability. I'm thinking, “I do hope that things are sustainable.” Right now it's gaining momentum but I hope that it is sustainable in the long term. P6, consumer representative
Reflexive monitoring	Use different methods and tools to measure success	… we send out an annual committee evaluation for everyone that attends the committee… So, we could add something in there around principles and evaluating that. P4, consumer engagement lead
		So I would really think that that enaction – tools that help people see how they're progressing, and can then translate that to, “I can see this change.” Either in behavior, in commitment, in the way decisions are being made, the way people are engaging in discussion. There's probably a variety of things that relate to those best practice principles. If people can see the difference that it's making or how it's actually happening in practice, that's a hugely empowering process. P11, policymaker
	Measure both short‐ and long‐term outcomes	P1: I think you'd probably have to do like a pre‐survey, and then implement the principles, and then evaluate after that.
		Interviewer: Okay, and would it be different if you were measuring short‐term versus long‐term outcomes, do you think?
		P1: You would probably do multiple evaluations, like you would have your initial, you implement 12 months later, you evaluate. A few years after that, you evaluate again. (P1, consumer engagement lead)
	Maintain flexibility in measurement	And I think that one of the things that we've learned as well is that everybody's at a different starting point, so you need to be able to cater for all those starting points in a very positive and empowering way. So I would really think that that enaction – tools that help people see how they're progressing, and can then translate that to, “I can see this change.” Either in behavior, in commitment, in the way decisions are being made, the way people are engaging in discussion. P11, policymaker

#### 3.2.1 Promote the Principles as Practical, Consumer‐Centered and Relevant

Participants stated that the BPP had several meaningful qualities that could motivate different interest‐holders to engage in its implementation. These qualities included that the BPP was relevant, useful, and likely to add value compared with existing partnership tools. Participants valued the practicality of the BPP and felt they helped to operationalize the concept of partnership which a consumer engagement lead described as “very fluffy.”

Consumer engagement leads felt the BPP resonated with their own experience, knowledge, and aspirations. Although the BPP were viewed as consistent with key government policy directions, they were viewed as sufficiently different and novel to add value. For example, the consumer‐centered focus of the BPP was viewed favorably when compared with Australia's National Safety and Quality in Health Care Standards [16].

#### 3.2.2 Champion the BPP as Useful for All Health Services, Regardless of Current Partnering Practices

Many participants believed the BPP offered examples of best practice that could help health services at all levels of partnering to improve their partnership approaches. For example, some health services with a higher level of maturity in partnering had already implemented practices such as having a majority of consumer representatives on some committees and/or consumers co‐leading meetings. However, other practices requiring more complex, structural change (such as consumer remuneration or flexible meeting times) may not have been implemented. In this way, the BPP provided a way of nudging health services toward improving even the most challenging aspects of partnering. Consumers also believed the BPP would help consumers feel more supported to participate.

#### 3.2.3 Integrate the BPP into Government Policies or Quality Standards

Participants perceived that health service leaders would be more motivated to implement the BPP if they were mandated as part of national or state government standards or policies. The requirement to implement the BPP to meet government standards would ensure external accountability for the implementation.

#### 3.2.4 Provide Evidence That the Benefits of the BPP Outweigh Implementation Costs

The policymaker interviewed perceived the government had many competing priorities and preferred simple approaches that could be implemented “in very small soundbites.” Government support for partnering initiatives, like the BPP, could be hampered if the costs of such initiatives outweigh the perceived benefits.

#### 3.2.5 Involve Health Service Leaders

Most participants felt it was important that the hospital board and executive staff endorsed and supported the BPP's implementation. From the consumer perspective, endorsement from the Chief Executive Officer was essential to successful implementation. Health service leadership would be more likely to endorse the implementation if the government provided funding to support BPP implementation.

#### 3.2.6 Designate (and Fund) Key Implementation Facilitators

Participants agreed that consumer engagement leads would most likely be the key facilitators of the BPP implementation. Consumer engagement leads would also need support from the organizational structures in which they were embedded, such as the Quality and Safety team. Although consumer engagement leads felt that health service executives generally had a positive and willing attitude toward improving partnering, funding was required to undertake the extra work needed to implement the BPP.

#### 3.2.7 Involve and Support Consumers Throughout the Implementation Process but Do Not Burden Them With Responsibility

Although both consumer representatives and consumer engagement leads perceived that health services (rather than consumers) should be responsible for BPP implementation, consumers felt they should be involved throughout the process.

#### 3.2.8 Promote the BPP Across the Health Service

To ensure organizational readiness, the BPP would need to be promoted across the health service to raise awareness and advocate for their adoption in health service committees. Consultation with staff and consumers prior to implementation was also recommended. Strategies to raise awareness of implementation included patient stories, staff training, and staff and consumer champions. Consumer representative P6 also suggested staff could potentially experience the BPP in action when consumers partnered with health service representatives on interview panels.

#### 3.2.9 Structure the Implementation and Ensure It Adds Value for Partnership Members

Consumer engagement leads perceived the implementation should be systematic and organized to ensure uptake of the BPP in committees. A structured approach could include a system for integrating the BPP into organizational strategies and policies alongside access to appropriate tools and resources to support understanding and use of the principles. Such tools could also add value to committee members by assisting them to fulfill evidence requirements for health service accreditation.

#### 3.2.10 Support Partnership Members to Implement the BPP

Participants described that improving partnerships would require practical and tailored support for the staff and consumers involved in committees. Organizational support systems also needed to be established to enable consumers to actively participate in committees. These systems included remuneration for consumers and support to manage vicarious trauma and resolve conflict. A suite of co‐designed, comprehensive resources (such as education sessions and written information) to accompany and support the BPP implementation was also recommended.

#### 3.2.11 Implement the BPP into Internal Policies and Procedures

To facilitate the integration of the BPP into routine practice, participants suggested they should be integrated into broader organizational policies such as the strategic plan and diversity and inclusion policies. Participants also expressed that the BPP should be incorporated into staff personal development plans and staff and consumer inductions to “help people to see it as ‘business as usual’ rather than something extra” (P11, policymaker).

#### 3.2.12 Use Different Methods and Tools to Measure Implementation Success

Consumer engagement leads suggested surveys or interviews with committee members (both staff and consumers) were ways to measure the implementation's success. Measurement could be embedded within existing internal feedback systems, such as annual surveys. Measurably demonstrating success was key to motivating interest‐holders to continue the implementation.

#### 3.2.13 Measure Short and Long‐Term Outcomes

Participants believed measuring both short‐ and long‐term outcomes of the implementation was necessary. This included checking whether specific changes and improvements resulting from the BPP implementation had been maintained.

#### 3.2.14 Maintain Flexibility in Measurement

Participants perceived that measurement of success should be flexible to ensure that health services at different stages of partnering with consumers could show improvements. Quantitative measurements of implementation effectiveness could also be useful.

## 4 Discussion

The BPP's capacity to build coherence among consumers and consumer engagement representatives demonstrated its relevance as a tool. Understanding the relevance of research is key to improving its translation into practice [17]. The findings showed that participants could understand how the BPP differed from existing resources and were considered valuable in improving partnering practices. Participants also reported on the flexibility of the BPP as a tool, suggesting it could be used by hospitals at different partnering maturities. Given the BPP directly resulted from engagement of the review's interest‐holder panel in the original Cochrane QES, these findings support previous claims that co‐produced evidence is more relevant to interest‐holders [5]. Specifically, the BPP may have facilitated knowledge translation because they provided “suitable packaging” for the review results [18]. Participants may also have found the BPP useful in translating evidence into practice because it removed the need to critically appraise and interpret individual review findings and develop their own tool [18].

The study showed that there were several additional factors that would affect whether the co‐produced BPP could be implemented into practice, echoing previous research suggesting that partnering with consumers will not improve without implementation guidance and support [19]. The findings demonstrated that interest‐holder engagement in implementing the BPP would be facilitated if the tool were integrated into government policies, designated funding were provided, and the benefits of implementation outweighed the real or perceived costs. These facilitators indicate that successful BPP implementation will require support not only at the micro (individual) level, but also from meso‐ (systems or organizations) and macro‐ (economic and political) levels [18]. These findings mirror previous studies about implementing partnering approaches that show policy and organizational support are necessary to facilitate improvement [20, 21, 22].

To engage interest‐holders in collective action (doing the work of implementing the BPP), the findings showed the implementation needed to be structured, supported by sufficient resources, and integrated into internal policies. Similar factors have been identified in implementing other initiatives to improve partnering with consumers [21, 22, 23] and evidence implementation more generally [18].

Finally, to help interest‐holders make judgements about the BPP's utility and effectiveness during implementation, the need for flexible measurement, using different formats (surveys and interviews), aligned with short‐ and long‐term outcomes was emphasized. Furthermore, measurement of the BPP's implementation would need to capture impacts across meso‐, macro‐, and micro‐levels to understand how they influence each other to initiate, enable, and sustain change [24]. Tools for measuring the effectiveness of partnering approaches in health system planning and decision‐making are a known challenge and should be explored in future research [25].

### 4.1 Implications for Evidence Synthesis Teams

This study has shown the value of implementation science in promoting the uptake of a co‐produced evidence synthesis in practice. Based on the findings, we have devised strategies (summarized in Box 1) to help evidence synthesis teams engaging in co‐production to optimize the translation of their review findings into practice. While we recognize the adoption of these strategies will be influenced by resource constraints on the review team and co‐production partners, they represent an aspiration for review teams to better integrate evidence co‐production with implementation science.

Box 1: **Strategies for evidence synthesis teams to increase knowledge translation of co‐produced reviews.**

During review co‐production

- Co‐produce the review with interest‐holders (including policymakers and health service executives) who can identify potential macro‐ and meso‐level implementation challenges during the review.
- Co‐produce a tool (e.g., best practice principles) from the review findings that interest‐holders perceive will benefit translation of review results.

During dissemination

- Interest‐holders involved in the review can play a key role in communicating the findings, depending on the audience.
- Policymakers and health service executives can help to upscale implementation of results using macro‐structures (e.g., policies, regulations, and financial tools).
- Frame dissemination to policymakers and health service executives according to their specific needs

During implementation

- Collaborate with local interest‐holders during implementation to assist, rather than drive, implementation.

#### 4.1.1 During Co‐Production

To address potential upstream barriers to implementation of review findings, we recommend including health policy makers and health service leaders as interest‐holders in the co‐production process. These groups understand the broader context of the health system or health service and have the authority to implement change [26]. If they perceive implementation is not feasible in practice, they can provide expert guidance on whether a research project should be re‐designed or disinvested [26].

We recommend developing a practical tool (such as the BPP) as part of the review process to improve implementation. Although tool development in isolation is insufficient to ensure implementation, it helps to bypass the need for implementers to integrate individual review findings [27]. Interest‐holders involved in the co‐production process can provide vital guidance about the feasibility of such tools in practice.

#### 4.1.2 During Dissemination

Although knowledge translation is largely the responsibility of research teams, researchers may not necessarily be the best messengers to communicate with different audiences [27]. For example, in a policy context, a policymaker who participated in the co‐production process may have more credibility and authority than the research team to communicate the results to their colleagues [27].

Targeting communication to policymakers and health service executives may also be highly beneficial in helping to upscale the implementation of review findings. Policymakers and health service executives are positioned to use macro structures, such as policy, regulation, and financial tools, to institutionalize a new innovation throughout a whole system or service [28]. When communicating to these groups, previous research supports tailoring the key messages to the audience's needs [29]. Furthermore, the engagement of policymakers and health service executives will be facilitated when their organizations have a strong research knowledge infrastructure, including a positive climate for research use [30].

#### 4.1.3 During Implementation

Local interest‐holders, rather than researchers, are responsible for the implementation of research [31]. Although researchers cannot drive implementation, they can facilitate its success [31]. Researchers can establish collaborative relationships with local interest‐holders, provide presentations about the research, and help to package the research in ways suited to the local context. However, implementation will be more successful if local interest‐holders own and have autonomy in the process [31].

### 4.2 Future Research Agenda

To better understand and optimize the co‐production of evidence syntheses for implementation, process evaluations that explore how co‐production influences implementation success are needed. Ideally, these should be compared with process evaluations of evidence syntheses that have not been co‐produced to clarify the differences in implementation impact (if any).

### 4.3 Strengths and Limitations

This exploratory study was limited by a small sample size, which, like other qualitative studies, was a direct result of pragmatic considerations [32]. Next steps for this research could include broadening the sample to include health service executives and health practitioners. The strengths of the interview sample were the inclusion of consumer engagement leads and consumer representatives from a range of hospitals as well as a government policy maker. The research was further strengthened by the active guidance and involvement of the panel.

## 5 Conclusion

Using the findings of a Cochrane QES as a case study, this research shed light on the contributions of co‐produced evidence to knowledge translation. Facilitators of knowledge translation included the relevance of the evidence to consumers and consumer engagement leads, and the packaging of the evidence as a practical tool (developed during the review). However, the study showed that co‐produced evidence still required implementation science approaches to identify barriers to implementation. Strategies for evidence synthesis teams engaging in co‐production to optimize the implementation of their review findings are provided.

## Author Contributions

**Bronwen Merner:** conceptualization, writing – original draft, investigation, methodology, writing − review and editing, formal analysis, funding acquisition. **Louisa Walsh:** conceptualization, funding acquisition, writing – original draft, methodology, writing – review and editing. **Janet Jull:** conceptualization, funding acquisition, writing – review and editing, methodology. **Nora Refahi:** conceptualization, investigation, writing – review and editing, methodology. **Vasileios Tsialtas:** conceptualization, investigation, writing – review and editing, methodology. **Benjamin Shemesh:** conceptualization, investigation, writing – review and editing, methodology. **Mel Kotze:** conceptualization, investigation, writing – review and editing, methodology. **Rebecca Ryan:** conceptualization, investigation, funding acquisition, writing – review and editing, methodology, supervision.

## Ethics Statement

The project was approved by the La Trobe University Human Research Ethics Committee (number HEC23054).

## Conflicts of Interest

V.T. received a consumer stipend from La Trobe University for participating in panel meetings. L.W. has received a Gilead Independent Medical Grant ($30,000 AUD) and a grant from Swinburne University ($9660 AUD) via her institution for research related to consumer involvement in health care. R.R. is supported by a grant from the National Health and Medical Research Council to support Cochrane groups in Australia. The other authors declare no conflicts of interest.

## Supporting information

Supporting file 1 implementation plan.

Supporting file 2 interview guide.

COREQ Checklist.

## Data Availability

The authors have nothing to report.
