# Supplementary material for: Promoting the Implementation of Co‐Produced Cochrane Evidence: An Exploratory Study of Improving Partnering With Consumers
Source: Cochrane Evid Synth Methods. 2026 Feb 3;4(2):e70071. doi: 10.1002/cesm.70071 (PMC12865661; doi:10.1002/cesm.70071)
Supplement: Supplementary file 1 — Supporting file 1 implementation plan. [file CESM-4-e70071-s001.docx]

**Supplementary material 1:**

**Implementation plan for piloting the Best Practice Principles for Partnering with Consumers in a health service in Victoria, Australia**

Abbreviations:

- BPP: Best Practice Principles
- SCV: Safer Care Victoria

| **Normalisation Process Theory Mechanism** | **Potential challenge** | **Implementation strategy (from taxonomy)** | **Description** |
| --- | --- | --- | --- |
| **Coherence**  How do stakeholders make sense of the Best Practice Principles (BPP)?  How do stakeholders differentiate the BPP from other partnering resources? | Different stakeholders may perceive meaning and value of the BPP differently e.g. clinicians versus hospital executive | Educational materials | Tailor educational materials about the meaning and value of the BPP to different stakeholders, focusing on the most important drivers for each audience (e.g. emotional, financial/economic, quality improvement) |
|  | People may not understand how the BPP are different to other partnering tools and resources (e.g. Partnering in Healthcare Framework) | Educational materials | Map the BPP to the Partnering in Healthcare Framework to reinforce how the two resources complement each other. Market BPP as a practical way to achieve Partnering in Healthcare Framework objectives. |
|  | Target audience for BPP (those doing the work) will require tailored resources | Educational materials | Educational materials targeted to people in committees, consumer engagement staff about how to implement the BPP. |
|  | Stakeholders who are not directly involved in the implementation also need to understand the meaning and value of the BPP to increase awareness | Educational materials/  Educational meetings/  Local opinion leaders | Educational materials targeted to staff not directly involved in the implementation through involvement of consumer representatives at staff inductions and presentations. Presentations will cover impact of the principles. Staff champions are needed, beyond the consumer engagement leads in organisations, for example, chairs or co-chairs of committees. |
|  | BPP should be reflected in organisational culture more broadly, not only in partnerships with consumers | Organisational culture | Some organisations may require greater upskilling of key principles (such as equity, diversity, accessibility across all contexts) rather than just in committees.  Encourage examination of how other committees, not only those involving consumers, reflect the BPP. |
|  | Extend understanding of meaning and value of BPP beyond an individual organisation | Community of Practice | Develop a Community of Practice to share value and knowledge across the health sector. This will be an opportunity to share improvements and achievements as a result of applying the BPPs. But other strategies also needed to avoid simply “preaching to the choir”. |
| **Cognitive participation**  (Gaining engagement and buy-in to implementing the BPP) | Buy-in from government sources is needed to enhance credibility of the implementation of the BPP | Organisational culture | BPP could be integrated within SCV refresh of Partnering in Healthcare Framework, particularly within the actions and outcomes domains of the framework. Include patient and consumer stories to support the need for the BPP and how they can make a positive difference in practice. |
|  | Strong support from health service leadership team needed to facilitate implementation, particularly for aspects that have financial implications for health service e.g. remuneration | Organisational culture  Educational materials  Educational outreach visit | Focus communications on how the BPP can help solve specific problems (e.g. stronger partnerships will lead to more person-centred decision-making). Also align with existing frameworks e.g. National Safety and Quality in Health Service Standard 2 (Partnering with Consumers) as well as Safer Care Victoria Partnering in Healthcare Framework. Communicate the “so what?” of implementing the BPP e.g. demonstrate impact of the BPP, provide clear evaluation measures that will show improvements.  Showcase other services that already align with the BPP to provide practical examples. |
|  | Engaging clinicians in a health service | Educational materials  Educational meetings  Educational outreach visits  Local consensus processes | Educational materials to demonstrate why the BPP are necessary. Should be targeted at new staff or departments who are starting to involve consumers. Include consumer stories.  Involve staff in co-designing the implementation, as well as Champions driving the implementation of the BPP |
|  | Engaging consumers | Local consensus processes | Involve consumers in co-designing implementation of BPP within the health service.  Establish a pathway for consumers receiving services to get involved in committees that align with their specific interests.  Encourage executive presence at consumer engagement/consumer representative events endorsing the BPP. |
| **Collective action**  (Implementing and embedding the BPP in hospital systems) | Involve consumers in the implementation of the BPP | Local consensus processes  Tailored intervention | Involve consumers in developing policy/procedure to support BPP in practice.  Involve consumers in sharing information about the implementation and impact of changes.  Provide funding for consumer engagement staff to connect consumers to staff seeking consumers to join a committee.  Establish roles for more experienced consumers and support them to advance their skills as consumer representatives (e.g. mentoring other consumers, board appointments, staff and consumer recruitment interviews, etc) as part of the BPP implementation |
|  | Consumers may not feel valued unless they are remunerated | Tailored intervention | Establish standardised remuneration system to demonstrate the value of consumer involvement, regardless of committee level. |
|  | Organisational uptake of the BPP is needed to ensure culture change and optimise impact | Organisational culture  Educational materials | Allocate funding to embed BPP in policies and procedures and governance documents.  Make adherence to BPP a requirement for establishing and reviewing key committee documents, such as terms of reference.  Integrate BPP into induction and orientation modules (for new staff? Or consumers or both?) as well as co-design/consumer engagement training.  Use consumer stories to demonstrate impact of BPP.  Use existing health service platforms to support information sharing about the BPP, and resources/tools to support implementation. |
|  | Implementation of the BPP may be perceived as overwhelming | Educational materials  Local consensus processes | Frame BPP as a process of development over time – not everything has to be done straight away.  Encourage flexible application of BPP but also emphasise the BPP work best as a package.  Use the approach that “it doesn’t matter where you start, but that you are moving towards a greater degree of consumer leadership”.  Emphasise small incremental changes that can be rapidly implemented and scaled  Develop “beginner”, “intermediate”, “advanced” examples of the application of the BPP to allow organisations/groups/committees to choose the principles that are right for their “stage” of experience with consumer involvement.  Encourage “partnering not punishing” e.g. what’s one small thing we can implement to help us move up the spectrum of involvement |
| **Reflexive monitoring**  (Evaluating the impact of implementing the BPP) | Maintaining the sustainability of the implementation | Educational meetings  Train the trainer  Continuous quality/local consensus processes | Incorporate the BPPs in mandatory staff and consumer training so everyone is on the same page, from the CEO down.  Slow, stepwise approach will assist with sustainability of change.  Embed into policies/processes.  Train the trainer programs about the BPP.  Have regular workshops with staff and consumers to assess how the implementation is progressing with the aim of always moving forward. |
|  | Evaluation approaches need to be inclusive, flexible and fit for purpose | Co-design  Audit and feedback | Evaluation approaches need to be developed with an understanding that transformation takes time.  Executives, clinicians and consumers should all be involved in design of the evaluation, including metrics used.  Develop a suite of evaluation measures so that health services can select measures appropriate to their current level of partnering. |
|  | Public reporting measures could be a mechanism for accountability | Public release of performance data | Public reporting on diversity and inclusion stats in health services could be a mechanism for accountability if tied to consumer engagement spaces (e.g. diversity of consumer representatives) |
|  | Quantitative metrics and qualitative evidence is needed to demonstrate impact | Audit and feedback | Quantitative outcomes that could be audited:   - Number of formal consumer partnerships and proportion of consumers on committees - Number of Committee ToR that refer to the BPP - Number of policies and procedures that refer to the BPP - Number of committees that have implemented the BPP - meeting flexibility e.g. mode of participation - demographic info (may help measure diversity) - remuneration of consumers in the organisation - reimbursement of consumer expenses e.g. parking fees - number of consumers who have undergone training in BPP (to be embedded in the Consumer induction or training modules) - opportunities for progression of consumer representatives (e.g. becoming a committee chair, paid employment in conducting consumer surveys, consumer lead, lived Experience roles, etc) - number of consumer representatives - consumer turnover   Feedback via interviews or surveys with clinicians and consumers (depending on type or volume of feedback required). Interviews could be with the Chair or Secretary of the Committee. Survey questions could be incorporated in employee survey and separate consumer survey.   - positive and negative impacts (including what has been achieved in the committee) - understanding of consumer role for all: consumer, committee chair and committee members - confidence to participate in committee - confidence to provide honest feedback - level of trust in the committee - level of involvement - satisfaction with meeting times and remuneration - satisfaction with consumer involvement in agenda setting and decision-making - Positive influence on consumer wellbeing, and impact on their mental health - barriers/enablers (including how issues identified in the implementation of the BPP are managed)   Safer Care Victoria is currently piloting an evaluation tool internally that maps to the Partnering in Healthcare Framework and will be part of Partnering in Healthcare refresh. Tool has extensive crossover with BPP. |
|  | Communicating impact of implementation | Educational meetings  Continuous quality  Educational materials | Consumer stories to support impact to staff.  Impact relayed to CEO, Executive Directors, Clinical Services Directors, Divisional Directors and Managers. This could be done via presentations from those driving the implementation and their Sponsors.  Regular newsletters about the positive impacts of the BPP |
