# Supplementary material for: Promoting the Implementation of Co‐Produced Cochrane Evidence: An Exploratory Study of Improving Partnering With Consumers
Source: Cochrane Evid Synth Methods. 2026 Feb 3;4(2):e70071. doi: 10.1002/cesm.70071 (PMC12865661; doi:10.1002/cesm.70071)
Supplement: Supplementary file 2 — Supporting file 2 interview guide. [file CESM-4-e70071-s003.docx]

**Supplementary material 2**

**INTERVIEW GUIDE**

- What are your views of the best practice principles as a way of improving partnerships with consumers in your service?
- Do you feel the principles represent a new way of working with consumers in your health service?
- Can you perceive a potential value of implementing the principles to your work? To your health service?
- How would implementing the principles affect your own way of working (if at all)? Your health service’s way of working?
- If the principles were implemented, who would be the key people *outside* your health service that would need to drive the implementation?
  - Australian Commission on Safety and Quality in Health Care (Standards)
  - Safer Care Victoria (PiH framework)
  - Other
- Who would be the key people *inside* your health service that would need to drive the implementation?
  - Do you perceive they would be open to supporting implementation of the principles and working in a new way?
  - Partnering “champions” or patient experience members that can help drive this work?
- How easily could you integrate the best practice principles into your current way of working with consumers?
  - Are there any existing frameworks/policies/principles that we can leverage to promote uptake and engagement of the best practice principles and the health service?
- How confident would you feel in the ability of staff in your health service to implement the best practice principles when engaging with consumers? Which groups or types of staff do you feel would have the most difficulty?
- Would the framing of the best practice principles make sense to your health service?
  - Follow up prompt: Are there any changes to the language that may be more suitable to a health service context rather a research setting?
- What are your perceptions about how implementing the best practice principles would disrupt relationships between health practitioners and consumers on health service committees?
- What resources and training would you need to implement the best practice principles in your work?
- What structures in your health service would help facilitate the integration of the principles?
- How would you evaluate whether the best practice principles had made a difference to your service?
  - In the short, medium and long term?
  - Do you have an existing method of monitoring and evaluating your partnering?
- How would you communicate the effectiveness of the principles in your health service?
- How would you sustain the implementation of the best practice principles over time?
  - Follow-up: How could we ensure that the implementation would continue if key people leave the organisation?

**Closing question:** Would you be interested in being contacted if there are opportunities in future to pilot the implementation of the best practice principles?
